# Supplementary figures and images for: Long Non-Coding RNA CRNDE Is Involved in Resistance to EGFR Tyrosine Kinase Inhibitor in EGFR-Mutant Lung Cancer via eIF4A3/MUC1/EGFR Signaling
Source: Int J Mol Sci. 2021 Apr 13;22(8):4005. doi: 10.3390/ijms22084005 (PMC8070547; doi:10.3390/ijms22084005)

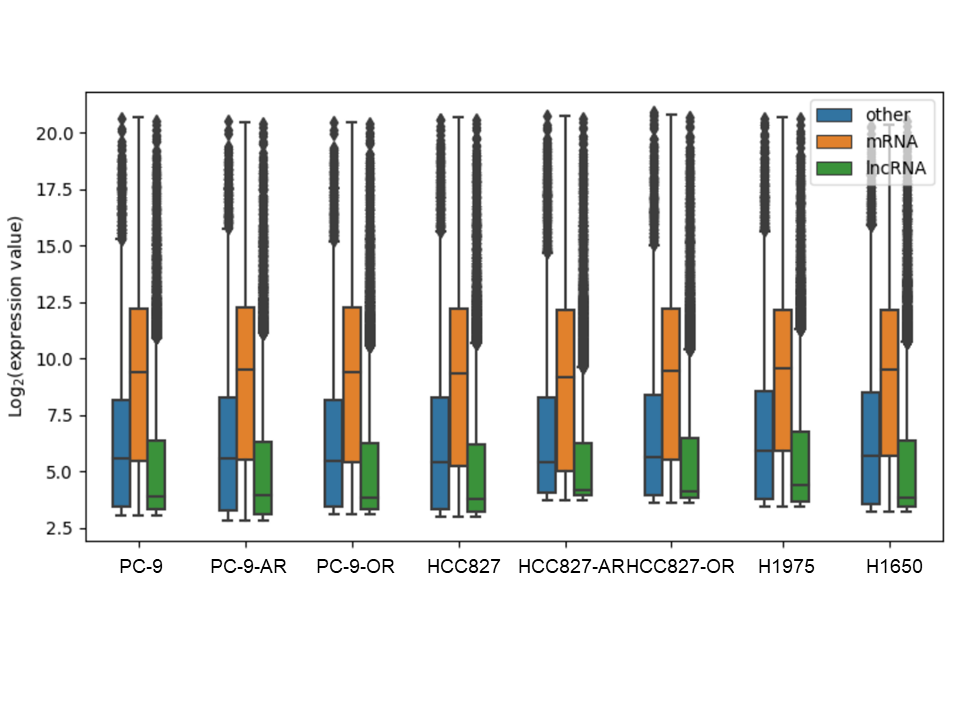

Supplement: Supplementary file 1 [file ijms-22-04005-s001.zip › Figure S1.TIF]
